# Immunomodulatory strategies for managing cytokine storms in chronic COVID: mechanisms, therapeutic targets, and clinical advances

**DOI:** 10.1097/MS9.0000000000004621

**Published:** 2025-12-16

**Authors:** Emmanuel Ifeanyi Obeagu

**Affiliations:** a Department of Biomedical and Laboratory Science, Africa University, Mutare, Zimbabwe; b The Department of Molecular Medicine and Haematology, School of Pathology, Faculty of Health Sciences, University of the Witwatersrand, Johannesburg, South Africa

**Keywords:** cytokine storm, immunomodulation, inflammation, long COVID, therapeutics

## Abstract

Chronic COVID, characterized by persistent symptoms following acute SARS-CoV-2 infection, is increasingly linked to sustained immune dysregulation, particularly cytokine storms that drive chronic inflammation and multi-organ complications. Understanding the mechanisms underlying cytokine dysregulation in chronic COVID is essential for developing effective therapeutic strategies aimed at restoring immune balance and mitigating long-term morbidity. This review critically examines current immunomodulatory strategies for managing cytokine storms in chronic COVID, including corticosteroids, cytokine-specific biologics, Janus kinase inhibitors, and emerging cell-based therapies. Additionally, the role of biomarker-guided precision medicine in personalizing treatment to optimize efficacy and safety is discussed. Challenges such as patient heterogeneity, timing and duration of therapy, and potential adverse effects are also addressed. Future research directions emphasize the need for robust clinical trials, novel therapeutic development, and integrated multidisciplinary care to improve patient outcomes. By tailoring immunomodulatory approaches based on individual immune profiles, it is possible to enhance the management of cytokine-driven inflammation in chronic COVID and advance the field toward more effective, personalized treatments.

## Introduction

The global COVID-19 pandemic, caused by the severe acute respiratory syndrome coronavirus 2 (SARS-CoV-2), has resulted in unprecedented morbidity and mortality worldwide. While the acute phase of COVID-19 has been the focus of intense clinical and scientific investigation, attention has increasingly turned to the long-term consequences of the infection. A significant subset of individuals who recover from the initial illness continue to experience a constellation of persistent symptoms, collectively termed chronic COVID or post-acute sequelae of SARS-CoV-2 infection. These symptoms can affect multiple organ systems and persist for weeks to months, substantially impairing quality of life and posing ongoing challenges to healthcare systems^[1,2]^. A hallmark of chronic COVID is the presence of chronic inflammation, often driven by persistent immune activation and cytokine dysregulation. The phenomenon of a cytokine storm – originally characterized in acute severe COVID-19 – refers to an excessive and uncontrolled release of pro-inflammatory cytokines, including interleukin-6 (IL-6), tumor necrosis factor-alpha (TNF-α), and interleukin-1 beta (IL-1β). While in the acute phase, cytokine storms can precipitate rapid clinical deterioration and organ failure, in chronic COVID, a more insidious, sustained cytokine imbalance contributes to ongoing tissue damage, fibrosis, and functional impairments across multiple organ systems^[3–5]^.

**HIGHLIGHTS**

- Long COVID involves persistent immune dysregulation and cytokine overproduction.
- Immunomodulators target key inflammatory pathways to restore immune balance.
- Therapies include corticosteroids, Janus kinase inhibitors, and biologics.
- Precision approaches improve patient-specific outcomes.
- Ongoing trials highlight promising clinical efficacy and safety profiles.

The pathophysiology of cytokine dysregulation in chronic COVID is complex and multifactorial. Potential mechanisms include residual viral antigens that persistently stimulate the immune system, failure of immune regulatory mechanisms such as regulatory T cells, and the development of autoimmune phenomena triggered by the infection. These processes lead to an amplified and prolonged inflammatory response, which manifests clinically as fatigue, dyspnea, cognitive dysfunction, and cardiovascular complications, among other symptoms^[6,7]^. Immunomodulatory therapies have become a cornerstone in managing cytokine storms in acute COVID-19, with agents such as corticosteroids and cytokine inhibitors demonstrating clinical benefit. However, the application of these therapies in chronic COVID presents unique challenges, including the need for long-term management strategies that balance efficacy with safety. Moreover, the heterogeneous nature of chronic COVID symptoms and immune profiles necessitates individualized treatment approaches that can be tailored to patient-specific inflammatory pathways and disease severity^[8,9]^. Recent advances have expanded the therapeutic landscape beyond conventional anti-inflammatory drugs. Targeted biologics, such as IL-6 and IL-1 receptor antagonists, offer more precise modulation of key cytokines involved in the inflammatory cascade. Janus kinase (JAK) inhibitors provide broad suppression of multiple cytokine signaling pathways, and emerging cell-based therapies like mesenchymal stem cells (MSCs) show promise for both immunomodulation and tissue repair. These novel approaches, combined with the growing field of biomarker-guided precision medicine, hold potential to improve outcomes by enabling tailored therapeutic regimens that address individual patient needs^[10,11]^.

## Aim

This review aims to comprehensively examine the immunomodulatory strategies currently employed and emerging for the management of cytokine storms in chronic COVID. It seeks to elucidate the underlying pathophysiology of chronic cytokine dysregulation, evaluate therapeutic interventions targeting immune imbalance, and explore the role of biomarker-guided precision medicine. Additionally, this article highlights the challenges faced in clinical practice and discusses future perspectives to optimize treatment outcomes for chronic COVID patients experiencing persistent inflammatory complications.

## Methods

This narrative review was developed using a structured and transparent approach to ensure comprehensive coverage of the evolving evidence on immunomodulatory strategies for managing cytokine storms in chronic COVID. A systematic search of the literature was conducted across PubMed, Scopus, Web of Science, and Google Scholar from January 2020 to December 2025. Search terms included combinations of “chronic COVID,” “post-acute COVID,” “cytokine storm,” “immunomodulation,” “immune dysregulation,” “nanoparticle therapy,” “immune checkpoint modulation,” and “precision immunology.” Reference lists of relevant articles and recent reviews were also screened to capture additional sources not retrieved during the primary search.

Eligible publications included peer-reviewed original research, clinical trials, meta-analyses, and high-quality narrative or systematic reviews focusing on mechanisms of immune dysregulation, therapeutic targets, or emerging treatment approaches for chronic or post-acute COVID inflammatory syndromes. Studies that addressed acute COVID without relevance to persistent immune activation were excluded. No restrictions were placed on study design or geographic region, although priority was given to studies presenting mechanistic insights or clinical implications.

Data extraction focused on recurring themes including cytokine network alterations, persistent innate–adaptive immune imbalance, targeted biologic therapies, advanced drug delivery systems, and precision medicine tools. The narrative synthesis approach allowed integration of mechanistic evidence with translational and clinical findings, emphasizing therapeutic relevance and emerging innovations. Discrepancies or overlapping concepts across studies were resolved through iterative comparison and refinement to ensure internal consistency throughout the manuscript.

### Pathophysiology of cytokine storms in long COVID

The cytokine storm in chronic COVID represents a sustained and dysregulated immune response that extends beyond the resolution of acute SARS-CoV-2 infection. Unlike the acute cytokine storm characterized by an overwhelming and rapid release of pro-inflammatory cytokines leading to severe respiratory distress and multi-organ failure, the cytokine dysregulation in Long COVID is more prolonged and insidious. This persistent inflammation contributes to chronic symptoms and ongoing tissue damage in various organ systems^[12,13]^. Several interconnected mechanisms underlie the pathophysiology of cytokine storms in chronic COVID. First, viral persistence or the presence of residual viral antigens in tissues can continuously stimulate innate immune receptors, such as toll-like receptors, maintaining a state of immune activation. This chronic antigenic stimulation prevents the normal resolution of inflammation and perpetuates the release of pro-inflammatory cytokines, including IL-6, tumor necrosis factor-alpha (TNF-α), and IL-1β^[14,15]^.

Second, there is evidence of immune regulatory failure, notably a dysfunction in regulatory T cells (Tregs) and other immunosuppressive pathways that ordinarily serve to limit excessive immune responses^[16]^. This impairment results in an unchecked pro-inflammatory milieu, which sustains the cytokine storm and exacerbates tissue injury. Additionally, chronic activation of macrophages and monocytes further fuels the production of inflammatory mediators, amplifying the immune cascade^[16]^. Third, autoimmunity and the generation of autoantibodies have been increasingly recognized as contributors to persistent inflammation in chronic COVID. The infection may trigger molecular mimicry or bystander activation, leading to immune responses directed against self-antigens. Such autoimmune phenomena can sustain cytokine production and prolong inflammatory damage, complicating the clinical course^[17]^. The downstream effects of sustained cytokine release include endothelial dysfunction, hypercoagulability, and fibrosis. Endothelial cells exposed to elevated cytokine levels express adhesion molecules and pro-coagulant factors, promoting vascular inflammation and microthrombosis. This contributes to persistent symptoms such as fatigue, dyspnea, and neurological impairment. Fibrotic remodeling, particularly in pulmonary and cardiac tissues, results from chronic inflammation and cytokine-induced activation of fibroblasts, leading to long-term organ dysfunction (Table 1)^[18,19]^.

**Table 1:** Pathophysiology of cytokine storms in long COVID

Pathophysiologic domain	Key mechanisms	Long COVID implications
Persistent innate immune activation	Sustained activation of monocytes, macrophages, and dendritic cells; chronic NF-κB signaling; dysregulated pattern-recognition receptor responses	Ongoing release of IL-6, TNF-α, IL-1β contributes to fatigue, musculoskeletal pain, neuroinflammation, and vascular dysfunction
Aberrant adaptive immune responses	Delayed recovery of T cell subsets, exhausted CD4/CD8 profiles, impaired regulatory T cell activity, persistent B cell hyperactivity	Prolonged inflammation, autoantibody formation, and reduced control of viral reservoirs
Viral persistence or residual antigen	Presence of viral RNA/proteins in tissue reservoirs (gut, CNS, endothelial tissue); impaired viral clearance	Continual immune stimulation leading to fluctuating or relapsing inflammatory symptoms
Endothelial dysfunction and microvascular injury	Endothelial apoptosis, glycocalyx degradation, microthrombi formation, complement overactivation (C3a, C5a)	Cardiovascular instability, POTS-like symptoms, cognitive impairment due to reduced tissue perfusion
Dysregulated cytokine networks	Imbalanced pro-inflammatory vs. anti-inflammatory cytokines; elevated IL-6, IL-8, IFN-γ, GM-CSF; impaired IL-10 responses	Multisystem inflammation, persistent fever, autonomic dysregulation, and organ-specific symptoms
Mitochondrial dysfunction and metabolic dysregulation	Impaired oxidative phosphorylation, increased ROS, metabolic reprogramming of immune cells	Fatigue, exercise intolerance, and chronic inflammatory milieu
Neuroimmune crosstalk abnormalities	Overactivation of microglia and astrocytes, blood–brain barrier disruption, neuroinflammatory cytokine spillover	“Brain fog,” headaches, sleep disturbances, mood alterations
Genetic and epigenetic susceptibility	Polymorphisms affecting cytokine expression, epigenetic modifications from acute infection, altered chromatin accessibility in immune cells	Heightened vulnerability to prolonged inflammatory states and poor recovery trajectory

### Immunomodulatory therapeutic strategies

The management of cytokine storms in chronic COVID necessitates a multifaceted immunomodulatory approach aimed at restoring immune balance, reducing inflammation, and preventing progressive tissue damage. Given the heterogeneous nature of the syndrome and the complex immune dysregulation involved, therapeutic strategies must be both targeted and adaptable to individual patient profiles. Current and emerging therapies can be broadly categorized into conventional anti-inflammatory agents, targeted biologics, small molecule inhibitors, and novel cell-based therapies^[20,21]^. Corticosteroids remain a cornerstone of immunomodulation due to their potent anti-inflammatory effects. They suppress multiple pro-inflammatory pathways by inhibiting the transcription of cytokines such as IL-6, TNF-α, and IL-1β. While corticosteroids have proven effective in reducing mortality in acute COVID-19 cytokine storms, their long-term use in Long COVID requires careful consideration of dosing and duration to minimize adverse effects such as immunosuppression, hyperglycemia, and osteoporosis^[22]^.

Cytokine-specific biologics have emerged as promising agents to precisely target key mediators of inflammation. Monoclonal antibodies against IL-6 receptor (e.g. tocilizumab), IL-1 receptor antagonists (e.g. anakinra), and TNF-α inhibitors are currently being explored in clinical trials for chronic COVID^[22]^. These agents offer the advantage of selectively dampening the cytokine cascade without broadly suppressing the immune system, potentially reducing the risk of secondary infections^[23]^. JAK inhibitors represent another class of targeted immunomodulators that interfere with intracellular signaling pathways essential for cytokine receptor signaling. By inhibiting JAK enzymes, these drugs modulate multiple cytokines simultaneously, offering a broad-spectrum anti-inflammatory effect. Early evidence from acute COVID-19 suggests potential benefits of JAK inhibitors such as baricitinib, but their safety and efficacy in chronic inflammatory states like chronic COVID warrant further investigation^[24,25]^.

Cell-based therapies, particularly mesenchymal stem/stromal cells (MSCs), are gaining attention due to their immunoregulatory and regenerative properties. MSCs can modulate immune responses by promoting anti-inflammatory cytokines, inhibiting pro-inflammatory pathways, and facilitating tissue repair^[26]^. Preliminary studies indicate that MSC therapy may reduce inflammation and improve functional outcomes in post-viral syndromes, including chronic COVID, though larger clinical trials are necessary to confirm these effects^[27]^. Beyond pharmacological interventions, supportive care and adjunctive therapies such as physical rehabilitation, nutritional support, and psychological counseling play essential roles in comprehensive management. Given the complexity of chronic COVID, a multidisciplinary approach integrating immunomodulation with symptom-targeted therapies is crucial for optimizing patient outcomes (Table 2)^[28]^.

**Table 2:** Immunomodulatory therapeutic strategies

Strategy	Mechanism of action	Clinical evidence	Considerations/limitations
Corticosteroids	Broad immunosuppression	Some symptom reduction in post-acute COVID	Risk of secondary infections, metabolic side effects
Cytokine-specific biologics	IL-6 (tocilizumab), IL-1 (anakinra), TNF-α inhibitors	Clinical trials ongoing	High cost, infection risk, limited long-term data
Mesenchymal stem cells (MSCs)	Promote anti-inflammatory cytokines, inhibit pro-inflammatory pathways, facilitate tissue repair	Early-phase trials suggest improved lung function	Cell source variability, regulatory hurdles
Targeted nanoparticle delivery systems	Deliver anti-inflammatory agents directly to immune or tissue targets	Preclinical models promising	Translational research ongoing, bioavailability concerns
Immune checkpoint modulation	Modulates T cell exhaustion and restores immune homeostasis	Preclinical studies; emerging clinical trials	Potential risk of immune overactivation, requires precise monitoring
AI-driven therapy personalization	Uses immune profiling and predictive modeling to tailor therapy	Pilot studies in other inflammatory diseases	Requires integration of robust datasets and clinical validation

### Biomarker-guided precision medicine

The heterogeneity of immune responses and clinical presentations in chronic COVID necessitates a precision medicine approach to immunomodulatory therapy. Biomarker-guided strategies leverage measurable indicators of immune activation and inflammation to tailor treatment regimens, optimize therapeutic efficacy, and minimize adverse effects. This approach moves beyond the traditional “one-size-fits-all” paradigm, enabling clinicians to identify patients who would benefit most from specific immunomodulatory interventions and to monitor treatment response dynamically^[29,30]^. Key biomarkers implicated in cytokine storm pathophysiology include circulating levels of pro-inflammatory cytokines such as IL-6, TNF-α, and IL-1β, as well as acute phase reactants like C-reactive protein and ferritin. Elevated cytokine profiles may help stratify patients with ongoing immune dysregulation and guide initiation or escalation of targeted therapies. For example, high IL-6 levels may indicate potential responsiveness to IL-6 receptor antagonists, whereas elevated IL-1β might suggest benefit from IL-1 blockade^[31,32]^.

**Figure 1.**
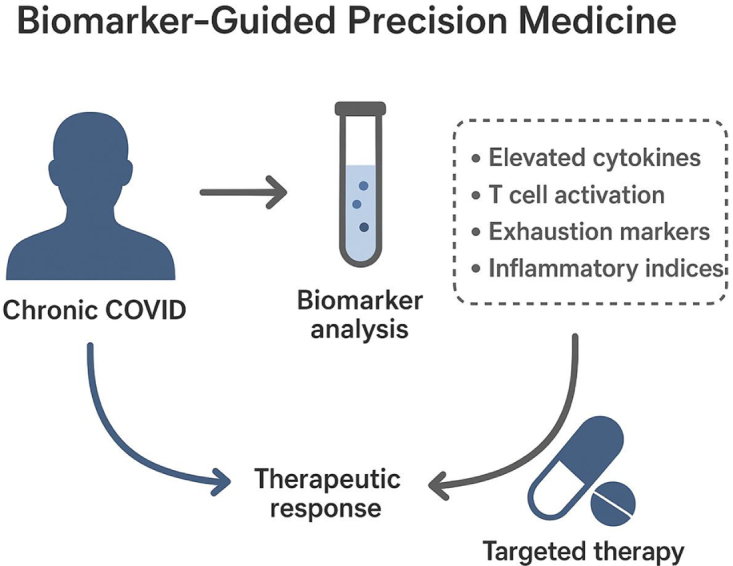
Biomarker-guided precision medicine.

In addition to cytokines, emerging biomarker panels incorporating immune cell phenotyping, gene expression profiles, and soluble receptor levels offer deeper insights into the immune landscape of chronic COVID patients. Advanced technologies such as single-cell RNA sequencing and proteomics enable the identification of specific immune cell subsets involved in persistent inflammation, including activated macrophages and exhausted T cells. These data can inform precision targeting of dysregulated pathways and facilitate the development of novel therapeutic agents^[33–35]^. Biomarkers also play a critical role in monitoring treatment efficacy and safety. Serial measurements allow for dynamic assessment of cytokine levels and inflammatory markers, helping to guide dosage adjustments, therapy duration, and early detection of potential immunosuppressive complications. Integrating biomarker data with clinical parameters improves decision-making and supports a more personalized, adaptive treatment approach (Fig. 1)^[36–38]^.

### Emerging therapeutic directions in immunomodulation for chronic COVID

Recent advances in immunology and biomedical engineering have opened new avenues for managing persistent cytokine dysregulation in chronic COVID. Beyond conventional anti-inflammatory agents and biologics, several innovative therapeutic strategies are being explored to achieve more precise, durable, and individualized immune modulation.

### Targeted nanoparticle delivery systems

Nanotechnology is increasingly being leveraged to deliver anti-inflammatory and immunomodulatory molecules with enhanced precision. Nanoparticle carriers, including lipid-based, polymeric, and biomimetic platforms, allow targeted delivery of therapeutic agents directly to inflamed tissues or specific immune cell populations. This targeted approach reduces systemic toxicity and enhances therapeutic efficacy. In preclinical models, nanoparticles engineered to transport corticosteroids, siRNA targeting pro-inflammatory cytokines, or small-molecule inhibitors have demonstrated the ability to suppress cytokine release while preserving essential immune function. Early-stage research in chronic COVID is examining nanoparticles capable of modulating macrophage activation, inhibiting inflammasome signaling, or stabilizing endothelial function, offering the potential for more refined control of hyperinflammation^[39,40]^.

### Immune checkpoint modulation

Immune checkpoint pathways that regulate T cell activation and exhaustion are emerging as potential therapeutic targets in chronic COVID, where prolonged immune activation coexists with dysfunctional antiviral immunity. Agents that modulate programmed death-1, cytotoxic T lymphocyte–associated antigen 4, and other checkpoint molecules may help restore immune balance by reducing aberrant cytokine release while improving T cell function. Although checkpoint inhibitors are primarily used in oncology, interest is growing in their ability to reset dysregulated immune networks in chronic infectious and inflammatory conditions. Preclinical studies and small pilot investigations have suggested that selective checkpoint modulation could alleviate persistent immune exhaustion and improve cytokine regulation, though safety considerations remain paramount given the risk of triggering excessive immune activation^[41,42]^.

### AI-assisted precision immunotherapy

Artificial intelligence and machine learning tools are increasingly being integrated into chronic COVID research to improve patient stratification and personalize treatment. AI-driven profiling can analyze complex datasets spanning cytokine signatures, genomic markers, metabolic profiles, and clinical phenotypes to identify patient-specific drivers of inflammation. This level of precision enables more targeted selection of immunomodulatory therapies, such as choosing between IL-6 blockade, JAK inhibition, or Treg-enhancing interventions based on individualized immune patterns. Emerging predictive models are also being developed to forecast treatment response and identify subgroups at risk for refractory inflammation. As these technologies mature, AI-guided immunotherapy has the potential to shift chronic COVID management from broad empirical treatment toward a more tailored, mechanism-based approach^[43,44]^.

### Challenges

The management of cytokine storms in chronic COVID presents several significant challenges that complicate therapeutic decision-making and clinical outcomes. A foremost challenge is the inherent heterogeneity of chronic COVID itself, encompassing a broad spectrum of symptoms, severities, and organ system involvement. This variability makes it difficult to establish uniform treatment protocols and to identify which patients will benefit most from immunomodulatory therapies^[39,40]^. Another critical issue is the incomplete understanding of the precise immunopathological mechanisms driving chronic cytokine dysregulation in chronic COVID. Although emerging evidence implicates persistent viral antigens, immune regulatory failure, and autoimmunity, the relative contribution of each mechanism likely varies between individuals. This complexity hinders the identification of definitive therapeutic targets and complicates the timing and selection of immunomodulatory agents^[41,42]^.

The risk-benefit balance of immunosuppression also poses a significant challenge. Prolonged or inappropriate use of immunomodulatory drugs, such as corticosteroids or biologics, can increase susceptibility to opportunistic infections, reactivation of latent pathogens, and other adverse events. This is particularly relevant in chronic COVID patients, who may have underlying comorbidities or immune dysfunctions that amplify such risks. Careful patient monitoring and judicious therapy adjustment are therefore essential but can be resource-intensive^[43]^. Biomarker limitations further impede optimal management. While numerous inflammatory markers have been proposed, standardized, widely accessible, and validated assays for guiding therapy remain limited. Variability in laboratory methods and lack of consensus on threshold values reduce the clinical applicability of biomarker-guided treatment strategies. Additionally, many biomarkers reflect systemic inflammation but may not fully capture tissue-specific immune dysregulation that drives localized symptoms^[44]^.

Another challenge is the scarcity of robust clinical trial data specifically addressing immunomodulatory interventions in chronic COVID. Most current evidence is extrapolated from acute COVID-19 or other chronic inflammatory diseases, limiting confidence in treatment efficacy and safety. The diverse and evolving nature of chronic COVID complicates trial design, recruitment, and outcome measurement, further delaying evidence generation^[45]^. Socioeconomic and healthcare disparities influence access to advanced immunomodulatory therapies and biomarker testing, particularly in low-resource settings. This inequity risks widening the gap in chronic COVID management and outcomes globally. Addressing these challenges requires concerted efforts in research, clinical innovation, and health policy to ensure equitable and effective care^[46]^.

### Future perspectives

The evolving understanding of cytokine dysregulation in chronic COVID opens promising avenues for the development of more effective and personalized immunomodulatory therapies. Future research is expected to focus on elucidating the molecular and cellular mechanisms that sustain chronic inflammation, with the aim of identifying novel therapeutic targets that can precisely modulate pathogenic immune responses without compromising host defense^[47]^. Advancements in multi-omics technologies – integrating genomics, transcriptomics, proteomics, and metabolomics – will be instrumental in unraveling the complex immune networks involved in chronic COVID. These comprehensive datasets will facilitate the discovery of new biomarkers and immune signatures that can guide patient stratification, prognosis, and individualized treatment plans, ultimately improving clinical outcomes^[48]^. The development of next-generation biologics and small-molecule inhibitors with improved specificity and safety profiles holds significant potential. Innovations such as bispecific antibodies, engineered cytokine traps, and selective JAK inhibitors tailored to chronic COVID pathophysiology may offer more effective control of cytokine storms while minimizing systemic immunosuppression and adverse effects^[49]^.

Cell-based therapies, including MSCs and regulatory T cell (Treg) adoptive transfer, represent a frontier for immunomodulation and tissue repair. Ongoing and future clinical trials will clarify their role and optimize protocols for their safe and efficacious use in chronic COVID patients with refractory inflammation and organ damage. Digital health technologies, such as wearable devices and remote monitoring platforms, can facilitate real-time assessment of inflammatory biomarkers and clinical parameters, enabling dynamic treatment adjustments and early detection of disease flares. Integrating artificial intelligence and machine learning algorithms may further enhance predictive modeling and decision support in personalized immunotherapy^[50]^. Addressing disparities in access to advanced diagnostics and therapeutics will be critical for ensuring equitable care. Strengthening healthcare infrastructure, fostering global collaborations, and implementing cost-effective strategies will be essential to translate scientific advances into broad clinical benefit^[51]^.

## Conclusion

Cytokine storms represent a critical pathogenic feature in chronic COVID, driving persistent inflammation and multi-organ dysfunction. Effective management requires a comprehensive understanding of the complex immune dysregulation underlying this syndrome. Immunomodulatory therapies, including corticosteroids, cytokine-specific biologics, JAK inhibitors, and cell-based treatments, offer promising avenues to restore immune balance and mitigate long-term sequelae. Biomarker-guided precision medicine is pivotal for tailoring interventions to individual patient profiles, optimizing therapeutic efficacy, and minimizing adverse effects. However, challenges such as heterogeneity of disease manifestations, incomplete mechanistic insights, and limited clinical trial data continue to impede standardized treatment approaches.

## Data Availability

Not applicable.
